# Assessment of Knowledge and Communication Regarding Complementary and Alternative Medicine Among Physicians in Madinah City

**DOI:** 10.7759/cureus.61307

**Published:** 2024-05-29

**Authors:** Omaymah Y Aljardi, Salma Alalawi, Afnan Z Aljohani, Ahmed A Humaida, Omran S Hashim, Ayat R Abdullah

**Affiliations:** 1 Department of Family Medicine, Prince Mohammed Bin Abdulaziz Hospital, Madinah, SAU; 2 Department of Internal Medicine, Prince Mohammed Bin Abdulaziz Hospital, Madinah, SAU; 3 Department of Family Medicine, Madinah Family Medicine Academy, Madinah, SAU; 4 Department of Emergency Medicine, Hayat National Hospital, Madinah, SAU; 5 Department of Family and Community Medicine, Taibah University, Madinah, SAU

**Keywords:** complementary and alternative medicine, madinah city, knowledge, communication, alternative, complementary

## Abstract

Introduction: Despite the recognition of the importance of physician knowledge and physician-patient communication about the use of complementary and alternative medicine (CAM), few studies have explored this issue. Therefore, this study aims to assess physicians' knowledge and physician-patient communication regarding CAM.

Methods: A cross-sectional study was conducted among physicians working at governmental hospitals and primary healthcare centers in Madinah, Saudi Arabia. The data collection tool was a validated English language questionnaire distributed using social media platforms. The questionnaire included sections to assess physician knowledge and communication about CAM.

Results: Of the 373 completed questionnaires, around 151 (40.5%) of the respondents stated that they have a poor level of knowledge about CAM, and 272 (72.9%) need to gain additional knowledge to properly counsel patients on CAM. Medical journals were the main source of knowledge about CAM. There were 121 (32.4%) physicians who believed that <20% of their patients use some form of CAM, and 180 (48.3%) believed that <20% of their patients spontaneously reported their CAM use without prompting or direct questioning. Around 180 (48.3%) of physicians believed that they asked <20% of their patients about using CAM. Regarding barriers that limit communication with the patient about CAM, the highest percentage was insufficient knowledge about CAM (137, 36.7%).

Conclusion: The study showed that a significant number of physicians lack the appropriate knowledge about CAM and most of them agreed to gain additional knowledge to properly counsel their patients. Further research is needed to evaluate physicians' knowledge about CAM using a more objective method.

## Introduction

Complementary and alternative medicine (CAM) is defined by the National Center for Complementary and Alternative Medicine (NCCAM) as “a group of diverse medical and healthcare systems, practices, and products that are not generally considered part of conventional medicine” [1]. In North America, the prevalence of CAM use by children, youth, and adults is well documented and varies widely (1.8-70%) [2]. There are five main types of CAM: alternative medical systems, biological therapies, body-based manipulation therapies, mind-body interventions, and energy therapies [3]. For centuries, CAM was recognized as a mainstream healthcare option for treating primary healthcare needs. The combination of CAM and conventional medicine is generally known as "integrative medicine," which emphasizes the biopsychosocial and spiritual aspects of the individual [4].

Despite uncertainties about the effectiveness of most CAM therapies, many studies have revealed a high prevalence of CAM use among the Saudi population (33-93%) [5-8]. Saudi Arabians typically use CAM based on their religious beliefs. A study revealed that the Saudi population spends around 8.2 billion US$ on CAM [9]. The most common types of CAM used among the Saudi community include Holy Quran therapy (9-95%) and herbs (8-76%), and the most commonly used were the black seed (3-61%), honey (14-73%), and hijama (cupping) (5-45%) [5]. In contrast to Saudi Arabia, the most common forms of CAM used in the Western world include relaxation techniques, ginseng, chiropractic, osteopathy, massage, mineral supplements, and homeopathy [10,11].

Lack of physician knowledge and education regarding CAM resulted in limited physician-patient communication [12,13]. It is crucial to have appropriate physician-patient communication regarding CAM to avoid unwanted consequences such as potential drug-herb interactions and misattribution of therapeutic benefits or side effects [14]. In addition, it enhanced the doctor-patient relationship and increased patient satisfaction [13].

Although the importance of physician-patient communication regarding the use of CAM is well recognized, few studies have explored this issue [15]. Therefore, the current study aims to assess physicians' knowledge and physician-patient communication regarding CAM.

## Materials and methods

This cross-sectional study was conducted at Madinah government hospitals and primary healthcare centers in Saudi Arabia. We included all primary healthcare doctors and physicians who are residents of Madinah. Physicians who refused to participate were excluded.

The sample size was calculated using the OpenEpi calculator [16]. A minimal sample size of 350 participants was required to achieve 95% confidence and a 5% margin of error, given that the total population number was one million, with 50% expected frequency. However, the total number of participants in this study was 380. The sampling method was convenient. The data collection tool was a semi-structured self-filled questionnaire in the English language distributed during the period of 20th March to 20th July 2023 using social media platforms, mainly through official WhatsApp channels. The first section collects demographic data of the participants, including age, gender, nationality, job description, specialty, and years of experience. The questions in the second section assess physicians' knowledge about CAM, whether they feel sufficiently knowledgeable about CAM safety and efficacy, their willingness to gain additional knowledge about CAM, the main source of their knowledge, and their history of formal training in CAM. The third section evaluates the quality of communication between physicians and their patients, and finally, a question to assess barriers to communication. The questionnaire was validated and reviewed by a community medicine professor at Taibah University and a pilot study was performed on 30 physicians to assure validation. An informed consent was obtained from each participant included in the study.

Ethical approval was obtained from the College of Medicine Scientific Ethics Committee at Taibah University (IRB00010413), in accordance with the principles stated in the Declaration of Helsinki.

Statistical analysis was performed using SPSS (IBM Corp., Armonk, NY). Descriptive data were reported as percentages, frequencies, or mean ± standard deviation (SD). The chi-square test has been used to assess the relationship between two qualitative variables. P < 0.05 was considered significant.

## Results

A total of 380 participants responded, and seven incomplete questionnaires were excluded. Completed questionnaires were received from 373 physicians from Madinah hospitals and primary healthcare centers in Saudi Arabia.

Around 181 (48.5%) of the respondents were less than 30 years of age and the same proportion (201, 53.9%) was of females. The majority of them (299, 80.2%) were Saudi. Regarding job descriptions, 215 (57.6%) of the participants were resident doctors, and most of the respondents (182, 48.8%) had less than five years of experience. The participant’s specialties were internal medicine (130, 34.9%), family medicine (116, 31.1%), surgery (93, 24.9%), and emergency medicine (34, 9.1%) (Table 1).

**Table 1:** Demographics of physician survey participants (N = 373).

Characteristics	Category	N = 373	%
Age, years	<30	181	48.5
	30-40	108	29.0
	41-50	58	15.5
	>50	26	7.0
Gender	Female	201	53.9
	Male	172	46.1
Nationality	Non-Saudi	74	19.8
	Saudi	299	80.2
Job description	Consultant	102	27.4
	Specialist	56	15.0
	Resident	215	57.6
Experience, years	<5	182	48.8
	5-10	104	27.9
	11-20	85	15.5
	>20	29	7.8
Specialty	Emergency medicine	40	10.7
	Family medicine	116	31.1
	Surgery	81	21.7
	Internal medicine	136	36.5

Around 151 (40.5%) of the respondents stated that they have a poor level of knowledge about CAM. The majority of respondents (272, 72.9%) stated that they need to gain additional knowledge to properly counsel patients on CAM.

Medical journals were the main source of knowledge about CAM (139, 37.3%), followed by formal medical education (126, 33.8%). On the other hand, non-medical sources (87, 23.3%) and specialized workshops (21, 5.6%) were minor sources of knowledge about CAM among the participants. Only 78 (20.9%) of the respondents had received formal training. The form of training was continued medical education (21, 5.6%) and residency program (16, 4.3%), and the most reported form was university study (41, 11%) (Table 2).

**Table 2:** Self-reported level of knowledge and learning resources among physicians (N = 373). CAM: complementary and alternative medicine.

Item	N	%
Feel sufficiently knowledgeable about CAM safety or efficacy		
Agree	79	21.2
Disagree	151	40.5
Neutral	143	38.3
Do you need to gain additional knowledge to properly counsel patients on CAM?		
Agree	272	72.9
Disagree	34	9.1
Neutral	67	18.0
The main source of your knowledge about CAM		
As a part of formal medical education	126	33.8
Specialized workshop	21	5.6
Medical journals	139	37.3
General non-medical sources	87	23.3
Did you receive any formal training in CAM?		
Yes	78	20.9
No	295	79.1
Which form was the training?		
Continued medical education	21	5.6
Residency program	16	4.3
University	41	11.0
None	295	79.1

Examination of the aggregate survey responses regarding physician-patient communication and patient use of CAM revealed a disparity between the perceived frequency of CAM use and the perceived frequency of patients’ CAM use (Table 3).

**Table 3:** Survey items regarding physician-patient communication and patients' use of CAM. CAM: complementary and alternative medicine.

Items	0-20%, n (%)	21-40%, n (%)		41-60%, n (%)	61-80%, n (%)	81-100%, n (%)
What percentage of your patients do you think to use some form of CAM therapy?	121 (32.4)	108 (29.0)		98 (26.3)	43 (11.5)	3 (0.8)
What percentage of your patients spontaneously report their CAM use without prompting or questioning?	180 (48.3)	95 (25.5)		59 (15.8)	34 (9.1)	5 (1.3)
In what percentage of your routine patient encounters do you directly ask your patients about their use of CAM?	180 (48.3)	76 (20.4)		49 (13.1)	53 (14.2)	15 (4.0)

The highest percentage (121, 32.4%) of physicians believed that <20% of their patients use some form of CAM; in addition, the highest percentage (180, 48.3%) of physicians believed that <20% of their patients spontaneously reported their CAM use without prompting or direct questioning, and 180 (48.3%) physicians believed that they asked <20% of their patients about using CAM (Table 3).

Regarding barriers that limit the ability to talk to the patient about CAM, the highest percentage was insufficient knowledge about CAM (137, 36.7%) (Table 4).

**Table 4:** Physicians' perspectives regarding barriers to communication with the patient about CAM. CAM: complementary and alternative medicine.

Perspective	Believe that patients will not respond	Don’t believe in the importance	Insufficient knowledge	Insufficient time
	n (%)	n (%)	n (%)	n (%)
In your opinion, what are the barriers limiting your ability to talk with patients about CAM during office visits or hospitalizations?	33 (8.9)	135 (36.2)	137 (36.7)	68 (18.2)

For different cross-tabulations for the relationship between the level of knowledge and learning resources by gender, there is a statistically significant relationship between feeling sufficiently knowledgeable about CAM and gender (Table 5).

**Table 5:** Relationship between the level of knowledge, learning resources, and gender. CAM: complementary and alternative medicine.

Variables		Gender				Total		X^2^	P-value
		Female		Male					
		n = 201	53.9%	n = 172	46.1%	n = 373	100%		
Feel sufficiently knowledgeable about CAM safety or efficacy	Agree	49	24.4	30	17.4	79	21.2	15.11	0.001
	Disagree	63	31.3	88	51.2	151	40.5		
	Neutral	89	44.3	54	31.4	143	38.3		
Do you need to gain additional knowledge to properly counsel patients on CAM?	Agree	150	74.6	122	71.0	272	72.9	2.43	0.296
	Disagree	14	7.0	20	11.6	34	9.1		
	Neutral	37	18.4	30	17.4	67	18.0		
The main source of your knowledge about CAM	As a part of formal medical education	77	38.3	49	28.5	126	33.7	5.76	0.124
	Specialized workshop	47	23.4	40	23.3	87	23.4		
	Medical journals	69	34.3	70	40.7	139	37.3		
	General non-medical sources	8	4.0	13	7.5	21	5.6		
Did you receive any formal training in CAM?	No	161	80.1	136	79.1	297	79.6	0.061	0.806
	Yes	40	19.9	36	20.9	76	20.4		
Which form was the training?	Continued medical education	7	3.5	14	8.1	21	5.6	6.52	0.089
	Residency program	12	6.0	4	2.3	16	4.3		
	University	23	11.4	18	10.5	41	11.0		
	None	159	79.1	136	79.1	295	79.1		

The differences between genders in terms of training and learning resources about CAM were not statistically significant (P > 0.05) (Table 5).

Regarding the relationship between the level of knowledge and learning resources by specialty, Table 6 showed a statistically significant relationship between specialties in terms of feeling sufficiently knowledgeable about CAM, gaining additional knowledge about CAM, sources of knowledge about CAM, as well as type of training (P < 0.05). On the other hand, there is no statistical difference between the level of knowledge and learning resources and receiving formal training about CAM by specialty (P = 0.091).

**Table 6:** Relationship between the level of knowledge, learning resources, and specialty. CAM: complementary and alternative medicine.

Variables		Specialty						Total		X^2^	P- value
		Family medicine		Surgery		Emergency and internal medicine					
		n = 116	31.1%	n = 81	21.7%	n = 176	47.2%	n = 373	100%		
Feel sufficiently knowledgeable about CAM safety or efficacy	Agree	29	25.0	20	24.7	30	17.0	79	21.2	10.04	0.04
	Disagree	38	32.8	27	33.3	86	48.9	151	40.5		
	Neutral	49	42.2	34	42.0	60	34.1	143	38.3		
Do you need to gain additional knowledge to properly counsel patients on CAM?	Agree	99	85.3	50	61.7	123	69.9	272	72.9	15.92	<0.001
	Disagree	4	3.5	10	12.4	20	11.4	34	9.1		
	Neutral	13	11.2	21	25.9	33	18.7	67	18.0		
The main source of your knowledge about CAM	As a part of formal medical education	56	48.2	22	27.0	48	27.3	126	33.8	26.97	<0.001
	Specialized workshop	6	5.2	3	3.7	12	6.8	21	5.6		
	Medical journals	38	32.8	25	31.0	76	43.2	139	37.3		
	General non-medical sources	16	13.8	31	38.3	40	22.7	87	23.3		
Did you receive any formal training in CAM?	No	87	75	71	87.7	139	79	297	79.6	4.79	0.091
	Yes	29	25.0	10	12.3	37	21.0	76	20.4		
Which form was the training?	Continued medical education	5	4.3	2	2.5	14	8.0	21	5.6	15.88	0.014
	Residency program	11	9.5	1	1.2	4	2.3	16	4.3		
	University	14	12.1	7	8.6	20	11.4	41	11.0		
	None	86	74.1	71	87.7	138	78.3	295	79.1		

The specialty of family medicine has a relationship with the level of knowledge and learning resources, and most of them agree to receive more training and courses about CAM in comparison with other specialties like surgery, emergency, and internal medicine (85.3% versus 61.7% and 69.9%) (Table 6).

## Discussion

The findings of this study highlight the need for improved physician knowledge and communication about CAM. The study revealed that a significant number of physicians felt insufficiently knowledgeable about CAM safety or efficacy, which could limit their ability to effectively discuss CAM with their patients during office visits or hospitalizations. This lack of knowledge may also contribute to barriers to implementing CAM in clinical practice.

Approximately 40.5% of the respondents stated that they have a poor level of knowledge about CAM. This finding was more significant in the studies conducted in Bahrain and Kuwait [14,17] in which the majority of participants (78% and 50%, respectively) stated that their level of knowledge was poor. The majority of respondents (72.9%) stated that they need to gain additional knowledge to properly counsel patients on CAM. In comparison with a study conducted in Riyadh, there was no significant difference, wherein 85% of participant physicians agreed that they should have knowledge about CAM [15]. Among New Zealand healthcare professionals, 58% expressed interest in receiving further education on CAM [18].

The highest percentage of the participant physicians (32.4%) estimated that less than 20% of their patients use some form of CAM. Approximately 48% of physicians ask only 20% or less of their patients about their use of CAM. In another study, 68% of physicians believed that only 15% of their patients use complementary medicine, and 58% of those physicians asked their patients about it [19].

Medical journals (37.3%) and formal medical education (33.8%) were the main sources of knowledge about CAM. In a study conducted in Bahrain, general non-medical sources such as general reading (48.2%) and general media like TV and radio (34.2%) were the main sources of knowledge about CAM [14].

One of the barriers identified in the study was the insufficient time available for physicians to discuss CAM with their patients. This finding is consistent with previous research that was conducted at the integrative medicine program of the University of Texas to address the vital aspect of patient-physician communication regarding CAM use in the context of cancer care, which has shown that time constraints were a common barrier to effective physician-patient communication about CAM [20]. Physicians often have limited time during patient encounters, and discussing CAM may be perceived as time-consuming. Therefore, strategies should be implemented to optimize time management and facilitate discussions about CAM within the constraints of busy clinical settings.

Another barrier identified in the study was the belief among some physicians that patients will not respond positively to discussions about CAM. This finding is in line with previous studies that have shown varying levels of patient disclosure and communication about CAM use [12,13]. The study also found that insufficient knowledge about CAM was a significant barrier for physicians. This highlights the importance of incorporating CAM education into medical curricula and continuing medical education programs. By providing physicians with evidence-based information about CAM, they can make informed decisions and effectively communicate with their patients about the potential benefits and risks of CAM therapies.

According to Robinson & McGrail (2004), patients may hesitate to disclose their CAM use due to concerns about a lack of understanding from their healthcare providers, the belief that there is no need for their healthcare providers to know about their CAM use, and the fact that they were not asked about CAM use [21]. It is crucial for physicians to create a non-judgmental and supportive environment that encourages open communication about CAM use.

The study further revealed that the specialty of family medicine had a significant relationship with the level of knowledge and learning resources about CAM. Family medicine physicians were more likely to agree to receive additional training and courses about CAM compared to other specialties. This finding suggests that there may be variations in CAM knowledge and communication among different specialties, highlighting the need for targeted educational interventions to address gaps in knowledge and improve communication skills.

The results showed that there were differences in the level of knowledge about CAM between male and female physicians. Female physicians tended to have a higher level of knowledge about CAM compared to male physicians. This is similar to a previous study conducted in New Zealand, which indicated that female doctors were more likely to agree to learn more about CAM compared to male doctors, and were more likely to use CAM with their patients [22]. Female physicians appear to have better access to learning resources about CAM, as evidenced by their higher utilization of seminars, courses, and journals related to CAM compared to male physicians.

Our study had several limitations. First, the study was conducted in a single city in Saudi Arabia, which may limit the generalizability of the findings to other regions or countries. Second, it relied on self-reported data from physicians, which may be subject to recall bias.

## Conclusions

In conclusion, this study emphasizes the importance of improving physician knowledge and communication about CAM. It showed that a significant number of physicians lack sufficient knowledge of CAM. In addition, most of them believe they need to gain additional knowledge to properly counsel patients on CAM. By addressing barriers such as insufficient knowledge, time constraints, and patient reluctance, healthcare providers can enhance patient care and satisfaction. Continued efforts to integrate CAM education into medical training and provide accessible resources for physicians are essential for promoting evidence-based discussions about CAM with patients. Further research is needed to evaluate physicians' knowledge about CAM using a more objective method.
